# Investigations on juvenile fish excluder cum shrimp sorting device (JFE-SSD)

**DOI:** 10.1186/2193-1801-2-271

**Published:** 2013-06-21

**Authors:** Menothuparambil Ravunny Boopendranath, Puthra Pravin, Therodath Rajan Gibinkumar, Sarasan Sabu, Vettiyattil Ramakrishnan Madhu

**Affiliations:** Central Institute of Fisheries Technology, CIFT Junction, P.O. Matsyapuri, Cochin, 682 029 India; Marine Products Export Development Authority, MPEDA House, Panampilly Avenue, Cochin, 682 036 Kerala India; Central Institute of Fisheries Nautical and Engineering Training, Fine Arts Avenue, Cochin, 682 016 India

**Keywords:** Juvenile fish excluder cum shrimp sorting device, JFE-SSD, Bycatch reduction, Shrimp trawl

## Abstract

Penaeid shrimp is a major resource in India contributing about 7.4% of the total marine fish landings. They are mostly landed by small mechanized trawlers. Shrimp trawling generates large quantities of bycatch mostly consisting of juvenile fishes, due to use of small mesh size in codends of trawl nets. Juvenile Fish Excluder cum Shrimp Sorting Device (JFE-SSD) is a bycatch reduction device with an *in situ* sorting mechanism, which replaces the conventional codend in a trawl. The device was designed to catch shrimps and commercially important fish species using a specially designed oval sorting grid with appropriate bar spacing and dual codends. Shrimp sorting efficiency and bycatch exclusion characteristics of JFE-SSD attached to a 29.6 m shrimp trawl, was tested by experimental fishing along the coastal waters off Cochin, India. Out of a total of 317.07 kg of catch encountered in the JFE-SSD installed trawl, 58.22% was retained in lower codend, 17.53% in upper codend and 24.25%, mostly consisting of juveniles and sub-adults of finfishes and shellfishes, was excluded from upper codend. The mean CPUE registered for upper and lower codend were 7.23±1.04 SE and 5.84±0.96 SE kg h^-1^ respectively. The CPUE of shrimps retained in upper and lower codends were significantly different (Kruskal-Wallis test (1,62), *P*<0.001), but the mean CPUE for fishes did not vary significantly. The average escapement of shrimps and juvenile fishes from upper codend were 0.06±0.02 SE kg h^-1^ and 2.40±0.44 SE kg h^-1^ respectively. Significant differences in the length composition between upper and lower codends were noticed for *Megalaspis cordyla*, *Stolephorus waitei*, *Metapenaeus dobsoni* and *Parapenaeopsis stylifera.* The experiments demonstrated *in situ* sorting ability of the device and its potential to reduce the bycatch of juveniles and sub-adults in shrimp trawls.

## Background

Shrimp trawling is a major economic activity in India and elsewhere in tropical fisheries. Average annual landings of penaeid shrimps in India, during 2010–2011, has been 0.26 million tonnes which formed 7.4% of the total marine fish landings (CMFRI 2013). They are mostly landed by small mechanized trawlers. It is well known that the shrimp trawling is a non-selective fishing method resulting in significant qantities of bycatch and discards (Kelleher 2005; Boopendranath et al. 2008,2010; Pramod 2010). Different types of bycatch reduction technologies have been developed in the fishing industry around the world with a view to reduce the bycatch and discards from trawling (Broadhurst 2000; Kennelly and Broadhurst 2002; CEFAS 2003; Eayrs 2007; Kennelly 2007; Boopendranath et al. 2008,2010; Boopendranath 2009,2012; Pravin et al. 2011; Broadhurst et al. 2012; Suuronen et al. 2012). Fishermen in India and elsewhere in tropical fisheries do not accept complete exclusion of fish and cephalopods during shrimp trawling, due to economic considerations (Boopendranath et al. 2008). Finfish species, crabs and cephalopods contribute significantly to the fishermen’s income. In small-scale mechanized trawl fisheries of India, shrimp fishermen spend a lot of time for sorting the catch onboard after they are landed, which cuts into their productive fishing time (Boopendranath et al. 2008). The struggle of large fishes accumulated in codend tend to damage shrimps leading to reduction in quality of the shrimp catch (Salini et al. 2000).

A number of bycatch reduction devices have been developed in different parts of the world, for shrimp fisheries (Kendall 1990; Isaksen et al. 1992; Rogers et al. 1997; Brewer et al. 1998; Robins and McGilvray 1999; Broadhurst 2000; Kennelly and Broadhurst 2002; CEFAS 2003; Courtney et al. 2006; Criales-Hernandez et al. 2006; Eayrs 2007; Eayrs et al. 2007; Hannah and Jones 2007; Heales et al. 2008; Broadhurst et al. 2012). In the present innovation named Juvenile Fish Excluder cum Shrimp Sorting Device (JFE-SSD), exclusion of juveniles and the separation of the catch into shrimp and fish are facilitated, during shrimp trawling. The design concept of JFE-SSD has won the Runner-up prize in International Smart Gear Competition-2005 of World Wildlife Fund (WWF 2005). The aims of the present study were to (i) assess the general performance of the JFE-SSD by experimental fishing in the conventional shrimp trawling grounds, off Cochin, (ii) assess sorting characteristics due to the installation of the JFE-SSD and (iii) quantify the escapement of shrimps and juveniles of fishes, through upper codend of JFE-SSD.

## Results and discussion

### CPUE and catch composition in upper and lower codends of JFE-SSD

**Figure 1:**
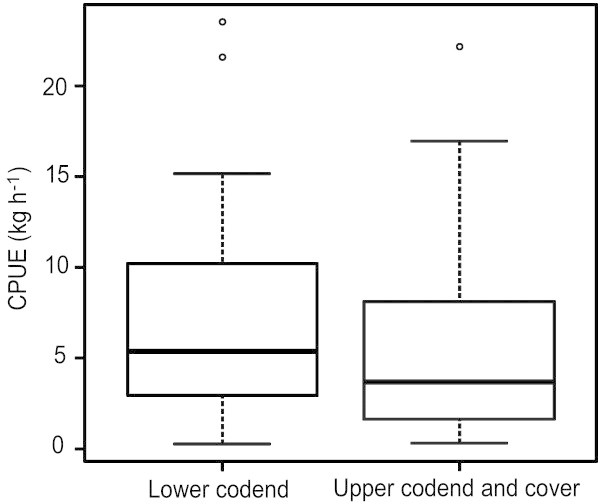
**Boxplot showing the mean CPUE (kg h**^**-1**^**) observed in upper and lower codends of trawl (Midline is the median and the boxes correspond to 25 and 75% of the values).**

Out of a total of 317.07 kg of catch landed during 31 hauls, 58.22% was retained in lower codend, 17.53% in upper codend and 24.25% mostly consisting of juveniles and sub-adults of finfishes and shellfishes was excluded from upper codend and retained in upper codend cover (Table 1). The mean catch per unit effort (CPUE) recorded for upper codend plus cover and lower codend were 5.84 ± 0.96 SE and 7.23 ± 1.04 SE kg h^-1^, respectively (Figure 1) and the difference in the means was not significant (F (1,60) =1.22, *P* = 0.27).

**Table 1:** **Species-wise catch distribution in upper codend, lower codend and upper codend cover and exclusion rates from upper codend in JFE-SSD installed operations**

	Encountered catch, kg	Lower codend, %	Upper codend, %	Upper codend cover, %	Exclusion rate from upper codend, %
**Finfishes**					
*Alepes kleinii*	3.35	47.01	4.93	48.06	48.06
*Ambassis ambassis*	4.33	42.38	11.89	45.73	45.73
*Anadontostoma chacunda*	1.15	28.70	56.52	14.78	14.78
*Arius jella*	2.67	28.09	51.87	20.04	20.04
*Cynoglossus arel*	0.86	23.98	14.62	61.40	61.40
*Cynoglossus macrostomus*	7.06	65.58	12.39	22.03	22.03
*Epinephelus diacanthus*	4.84	31.40	22.21	46.38	42.16
*Escualosa thoracata*	1.46	59.79	2.41	37.80	46.38
*Gerres erythrourus*	1.13	46.22	21.33	32.44	37.80
*Johnius borneensis*	7.63	46.85	24.64	28.51	19.53
*Johnius carouna*	0.78	14.10	57.69	28.21	32.44
*Johnius carutta*	2.23	18.88	54.38	26.74	28.51
*Lactarius lactarius*	1.10	30.14	16.89	52.97	28.21
*Leiognathus dussumieri*	1.00	42.21	10.05	47.74	26.74
*Leiognathus equulus*	1.03	65.37	4.88	29.76	52.97
*Eubleekeria splendens*	8.66	57.65	22.82	19.53	47.74
*Liza parsia*	1.02	32.84	14.71	52.45	52.45
*Megalaspis cordyla*	3.65	11.10	34.11	54.79	54.79
*Mugil cephalus*	2.71	82.29	2.21	15.50	34.42
*Opisthopterus tardoore*	1.58	40.63	57.14	2.22	19.70
*Otolithes ruber*	1.49	20.81	30.87	48.32	2.22
*Pampus argenteus*	3.18	0.00	100.00	0.00	3.28
*Pellona ditchella*	1.53	45.75	8.50	45.75	0.00
*Rastrelliger kanagurta*	8.93	30.52	32.53	36.95	3.14
*Sardinella albella*	1.79	46.78	17.93	35.29	33.54
*Sardinella longiceps*	43.64	37.03	15.96	47.00	36.95
*Scoliodon laticaudus*	0.35	0.00	100.00	0.00	35.29
*Secutor insidiator*	5.27	32.86	30.77	36.37	47.00
*Stolephorus waitei*	1.10	73.52	13.70	12.79	36.37
*Thryssa mystax*	3.37	35.91	29.38	34.72	12.79
*Trypauchen vagina*	0.85	5.88	76.47	17.65	34.72
**Shrimps**					
*Metapenaeus dobsoni*	49.67	95.62	1.57	2.80	2.80
*Parapenaeopsis stylifera*	6.47	97.74	0.43	1.82	48.32
*Penaeus monodon*	0.80	2.52	94.34	3.14	1.82
**Crabs**					
*Charybdis ferriatus*	0.88	17.71	61.14	21.14	21.14
*Doclea ovis*	9.57	30.93	26.91	42.16	29.76
*Portunus sanguinolentus*	8.95	25.21	41.25	33.54	45.75
**Stomatopods**					
*Oratosquilla nepa*	19.98	96.47	0.25	3.28	15.50
**Molluscan shells**	79.38	63.21	17.09	19.70	0.00
**Miscellaneous unidentified species**	11.71	43.21	22.37	34.42	17.65
**All species**	**317.07**	**58.22**	**17.53**	**24.25**	**24.25**

The retention of shrimps in upper and lower codends was 2.82% and 97.18%, respectively, which indicates good segregation of shrimps in the JFE-SSD (Table 2). The mean CPUE of shrimps in upper and lower codends was 0.11 ± 0.05 SE and 2.03 ± 0.43 SE kg h^-1^ respectively. The mean CPUE of shrimps retained in upper and lower codends of the trawl were significantly different (Kruskal-Wallis test (1,62), *P* < 0.001).

**Table 2:** ***In-situ*sorting effect on species groups due to installation of JFE-SSD**

Species groups	Catch (upper and lower codends), kg	Catch in lower codend, %	Catch in upper codend, %
All species	240.19	76.86	23.14
Shrimp species	55.40	97.18	2.82
Species other than shrimps	184.79	70.77	29.23

**Figure 2:**
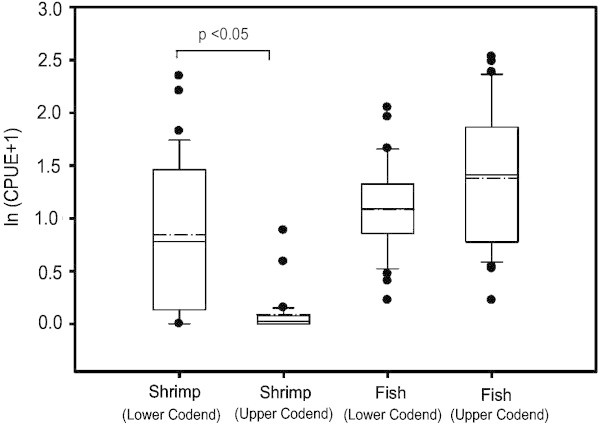
**Boxplot showing the mean CPUE (kg h**^**-1**^**) of shrimps and fishes caught in upper and lower codends of the trawl (Dotted line shows mean and the thick line is the median).**

The mean CPUE of finfishes in upper codend was 3.91 ± 0.59 SE kg h^-1^ contributing 66.95% of the total CPUE of finfishes. The mean CPUE for finfishes in lower codend was 2.26 ± 0.27 SE kg h^-1^, which formed 31.26% of the total CPUE. No significant difference was noticed in the mean CPUE of finfishes between upper and lower codends (Kruskal-Wallis test (1,62), *P* = 0.12) (Figure 2). The mean CPUE for total catch observed in the fishing ground with traditional codends has been reported to be 10.7 kg h^-1^ (Boopendranath et al. 2008) and, hence, it can be considered that there is no conspicuous difference in the catch rates due to installation of JFE-SSD.

### Bycatch exclusion characteristics of JFE-SSD

Species other than shrimps excluded through upper codend of JFE-SSD was 28.96% by weight. Among the species encountered, four species, *viz*., *Cynoglossus arel, Megalaspis cordyla, Lactarius lactarius* and *Liza parsia* showed exclusion rates in excess of 50%. Nineteen species, *viz*., *Otolithes ruber*, *Alepes kleinii*, *Leiognathus dussumieri*, *Sardinella longiceps*, *Epinephelus diacanthus*, *Pellona ditchella*, *Ambassis ambassis*, *Doclea ovis*, *Escualosa thoracata*, *Rastrelliger kanagurta*, *Secutor insidiator*, *Sardinella albella*, *Thryssa mystax*, *Portunus sanguinolentus*, *Gerres erythrourus*, *Leiognathus equulus*, *Johnius borneensis*, *Johnius carouna* and *Johnius carutta*, showed exclusion rates between 25 and 50% by weight. Species such as *Cynoglossus macrostomus*, *Charybdis ferriatus*, *Arius jella*, *Eubleekeria splendens*, *Trypauchen vagina*, *Mugil cephalus*, *Anadontostoma chacunda*, *Stolephorus waitei*, *Oratosquilla nepa*, *Penaeus monodon*, *Metapenaeus dobsoni*, *Opisthopterus tardoore*, *Parapenaeopsis stylifera* and molluscan shells, showed exclusion rates up to 25% by weight (Table 1). Two finfish species, *viz*., *Pampus argenteus* and *Scoliodon laticaudus* were not excluded through JFE-SSD.

**Figure 3:**
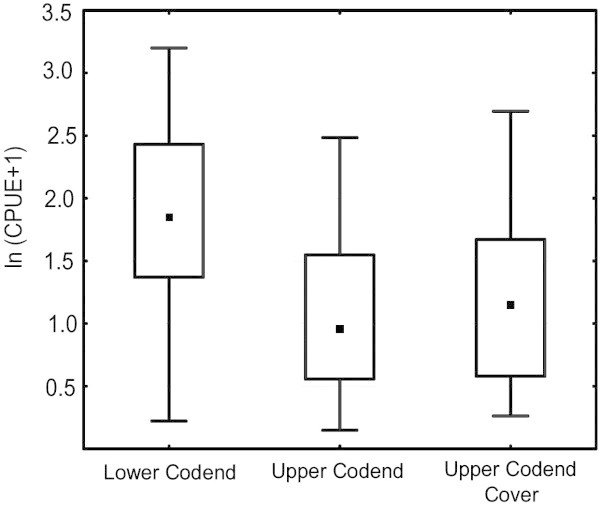
**Boxplot showing the mean ln(CPUE) (kg h**^**-1**^**) observed in the lower codend, upper codend and upper codend cover of the trawl installed with JFE-SSD (The small square in the box represents the median and the boxes correspond to 25-75% of the values).**

The mean escapement from upper codend, expressed as CPUE was significantly higher than zero in case of both fish and shrimp (t = 2.88, *P* = 0.11, df = 1). The average escapement of shrimps from the from upper codend was 0.06 ± 0.02 SE kg h^-1^ and the escapement of juvenile fish from upper codend was 2.40 ± 0.44 SE kg h^-1^ (Figure 3).

### Sorting characteristics of JFE-SSD

Out of a total catch of 240.19 kg in upper and lower codends, about 77% was retained in lower codend and the rest in upper codend. Of the retained catch other than shrimps (184.79 kg), 71% was retained in lower codend and 29% in upper codend. Out of a total catch of 55.40 kg of shrimps, about 97% was retained in lower codend (Table 2). *Pampus argenteus* and *Scoliodon laticaudus* was retained 100% in upper codend. Finfishes such as *Trypauchen vagina*, *Johnius carouna*, *Megalaspis cordyla*, *Johnius carutta*, *Anadontostoma chacunda*, *Arius jella*, *Portunus sanguinolentus, Otolithes ruber*, *Opisthopterus tardoore* and *Rastrelliger kanagurta* and crab species *Charybdis ferriatus* were retained in upper codend at rates exceeding 50%. *Penaeus monodon* also has shown preference to upper codend, though overall catch volume for this species was very low during the period of operations. Shrimp species such as *Parapenaeopsis stylifera* and *Metapenaeus dobsoni*; squilla *Oratosquilla nepa*; finfishes such as *Mugil cephalus*, *Escualosa thoracata*, *Leiognathus equulus*, *Alepes kleinii*, *Pellona ditchella*, *Stolephorus waitei*, *Cynoglossus macrostomus*, *Leiognathus dussumieri*, *Ambassis ambassis*, *Sardinella albella*, *Eubleekeria splendens, Sardinella longiceps*, *Liza parsia*, *Gerres erythrourus*, *Johnius borneensis*, *Lactarius lactarius*, *Cynoglossus arel*, *Epinephelus diacanthus*, *Thryssa mystax* and *Secutor insidiator*; crab *Doclea ovis*; and molluscan shells preferentially accumulated in lower codend (Table 3). The sorting effect was very pronounced in shrimp species. However, the sorting of finfish species was not very effective with the grid, probably due to prevalence of juveniles of finfishes. The device may need further optimization in terms of grid bar interspaces and opening to upper codend, to reduce the finfish catches in lower codend.

**Table 3:** **Sorting effect on trawl caught species in JFE-SSD**

Species	Catch (upper and lower codends), kg	Catch in lower codend,%	Catch in upper codend, %
*Oratosquilla nepa*	19.32	99.74	0.26
*Parapenaeopsis stylifera*	6.36	99.56	0.44
*Metapenaeus dobsoni*	48.28	98.38	1.62
*Mugil cephalus*	2.29	97.38	2.62
*Escualosa thoracata*	0.91	96.13	3.87
*Leiognathus equulus*	0.72	93.06	6.94
*Alepes kleinii*	1.74	90.52	9.48
*Pellona ditchella*	0.83	84.34	15.66
*Stolephorus waitei*	0.96	84.29	15.71
*Cynoglossus macrostomus*	5.51	84.11	15.89
*Leiognathus dussumieri*	0.52	80.77	19.23
Molluscan shells	63.75	78.71	21.29
*Ambassis ambassis*	2.35	78.09	21.91
*Sardinella albella*	1.16	72.29	27.71
*Eubleekeria splendens*	6.97	71.64	28.36
*Sardinella longiceps*	23.13	69.88	30.12
*Liza parsia*	0.49	69.07	30.93
*Gerres erythrourus*	0.76	68.42	31.58
*Johnius borneensis*	5.46	65.54	34.46
*Lactarius lactarius*	0.52	64.08	35.92
*Cynoglossus arel*	0.33	62.12	37.88
*Epinephelus diacanthus*	2.60	58.57	41.43
*Thryssa mystax*	2.20	55.00	45.00
*Doclea ovis*	5.54	53.48	46.52
*Secutor insidiator*	3.35	51.64	48.36
*Rastrelliger kanagurta*	5.63	48.40	51.60
*Opisthopterus tardoore*	1.54	41.56	58.44
*Otolithes ruber*	0.77	40.26	59.74
*Portunus sanguinolentus*	5.95	37.93	62.07
*Arius jella*	2.14	35.13	64.87
*Anadontostoma chacunda*	0.98	33.67	66.33
*Johnius carutta*	1.63	25.77	74.23
*Megalaspis cordyla*	1.65	24.55	75.45
*Charybdis ferriatus*	0.69	22.46	77.54
*Johnius carouna*	0.56	19.64	80.36
*Trypauchen vagina*	0.70	7.14	92.86
*Penaeus monodon*	0.77	2.60	97.40
*Pampus argenteus*	3.18	0.00	100.00
*Scoliodon laticaudus*	0.35	0.00	100.00
Miscellaneous species	7.60	65.89	34.11
**All species**	**240.19**	**76.86**	**23.14**

### Length based separation of species between upper and lower codends

**Figure 4:**
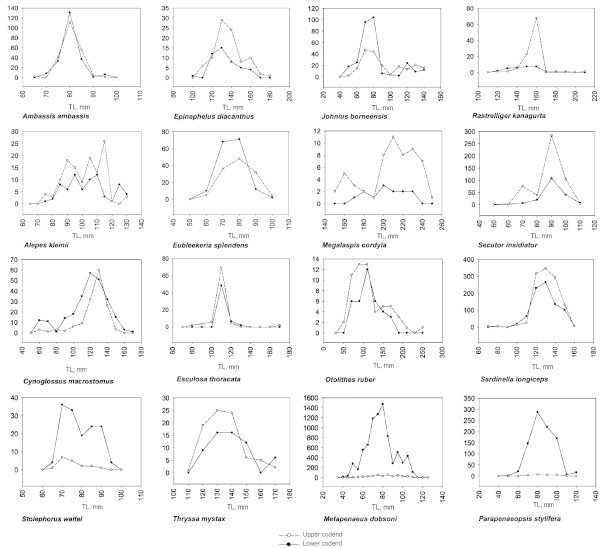
**Length based separation of species (numbers) between upper and lower codends.**

Anderson-Darling K-sample test was used to test the hypothesis of length-based separation of species in upper and lower codends. Significant difference in the length composition between upper and lower codends was noticed for *Megalaspis cordyla* (larger length groups were represented in upper codend) (AD = 5.89, *P* = 0.001, criteria = 0.7, df = 20) and *Stolephorus waitei* (larger length groups were represented in lower codend) (AD = 2.15, *P* = 0.04, criteria = 0.69, df = 16). In the case of shrimps, significantly higher catches and larger length groups were noticed in lower codend for *Metapenaeus dobsoni* (AD = 7.12, *P* = 0.001, criteria = 0.73, df = 36) and *Parapenaeopsis stylifera* (AD = 5.23, *P* = 0.003, criteria = 0.69, df = 16). Length based separation of other species, were not significant statistically. The length based retention and exclusion of selected species caught during the experiments are given in Figure 4.

### Selectivity characteristics of JFE-SSD

Different length classes of juveniles (71–110 mm TL) of *Alepes kleinii* were excluded through JFE-SSD at rates between 52 and 80%, during the field trials. Length classes of 71–100 mm TL was completely excluded in the case of *Escualosa thoracata,* while length classes from 101 to 120 mm TL were excluded at levels of 42-58% and length classes >120 mm TL were fully retained. Juveniles of *Otolithes ruber* in the length range of 51–170 mm TL showed exclusion rates of 40-68%. *Rastrelliger kanagurta* above 161 mm TL were retained while length classes of juveniles below 160 mm TL were excluded at levels of 77-90%. Juveniles of *Epinephelus diacanthus* below 110 mm TL were excluded, while length classes between 111 and 160 mm TL were excluded at levels from 46 to 75%. Length classes of *Cynoglossus macrostomus* above 151 mm TL were fully retained while those in 81–150 mm TL length class were excluded at levels from 13 to 54% and those in 71–80 mm length class was excluded at a level of 75%. Adult *Sardinella albella* in the length class of 156–160 mm TL were retained while length classes in the 126–155 mm range were excluded at levels ranging from 39 to 70%. Length classes of *Secutor insidiator* from 51 to 100 mm TL were excluded at rates ranging from 40 to 93%, while length class below this range (41–50 mm TL) was fully excluded. Length classes of *Stolephorus waitei* in the range of 61–90 mm TL were excluded at rates ranging from 4 to 16% while adult length class above this (91–96 mm TL) was fully retained. In the case of *Thryssa mystax*, length class 101–110 mm TL showed 100% exclusion while larger length classes (111–170 mm TL) showed exclusion rates of 25-68%.

The estimates of mean selection length (L_50_) and selection range in respect of *Ambassis ambassis*, *Cynoglossus macrostomus*, *Epinephelus diacanthus*, *Johnius borneensis*, *Lepturacanthus savala*, *Megalaspis cordyla, Sardinella albella, Sardinella longiceps, Secutor insidiator*, *Stolephorus waitei* and *Thryssa mystax* were derived (Table 4). Selectivity curves and mean selection lengths indicate that JFE-SSD is able to provide escape opportunities to juveniles and sub-adults. L_50_ values higher than length at first maturity (L_m_) values (Modayil and Jayaprakash 2003; Froese and Pauly 2011) indicate better exclusion opportunities for immature fishes below L_m_. L_50_ values in respect of *Ambassis ambassis* and *Thryssa mystax* were higher than L_m_ values reported. Length based analysis of catches shows that while considerable quantities of juveniles were excluded through upper codend, sizeable amount of fishes of smaller length groups were retained in lower codend. The escapement from upper codend was 2.40 ± 0.44 and 0.06 ± 0.02 kg h^-1^ respectively for fishes and shrimps. Considering the L_50_ values for the species studied, we conclude from the results that there has been considerable exclusion of juveniles from upper codend.

**Table 4:** **Selectivity parameters in respect of selected trawl caught species in JFE-SSD installed operations, during November–December 2005**

Species	L_25_ (TL, mm)	L_50_ (TL, mm)	L_75_ (TL, mm)	Selection range (TL, mm)	L_m_ (TL, mm)
*Ambassis ambassis*	59.44	83.86	108.27	48.83	55-75^a^
*Cynoglossus macrostomus*	73.92	211.25	348.58	274.65	NA
*Epinephelus diacanthus*	50.97	105.9	160.83	109.86	210-377^a^
*Johnius borneensis*	46.42	91.27	136.11	89.68	140-160^a,b^
*Lepturacanthus savala*	50.05	75.02	99.99	49.94	418-750^a^
*Megalaspis cordyla*	95.67	150.6	205.53	109.86	250^b^
*Sardinella albella*	109.35	140.74	172.13	62.78	90^b^
*Sardinella longiceps*	17.04	126.90	236.76	219.72	150-162^b^
*Secutor insidiator*	64.63	125.67	186.70	122.07	67^a^
*Stolephorus waitei*	27.18	43.34	59.49	32.31	81-84^a^
*Thryssa mystax*	98.55	156.37	214.19	115.64	130^a^

Source: ^a^Modayil and Jayaprakash (2003); ^b^Froese and Pauly (2011).

We have observed that a standard commercial trawl, used in the small-scale mechanized sector, can be fitted with JFE-SSD, by replacing conventional codend, in about half an hour, in field conditions. Clogging of the grid bar interspaces due to plastic refuse or decaying vegetation when they are prevalent in the fishing grounds has been observed to influence the efficiency of sorting and target catch retention, as they tend to block the interspaces of grid bars (unpublished observations).

Results indicate that JFE-SSD has excellent juvenile bycatch reduction and pre-sorting capabilities. During JFE-SSD operations off southwest coast of India, we have observed a reduction in bycatch by 29% with shrimp loss of less than 3%. The device has shown potential to reduce the bycatch of juveniles of finfishes, shrimps, crabs and cephalopods and small sized fishes of low commercial value, which will support sustainability of the resources and protection of biodiversity. The fishermen will be able to retain large fishes, which may enhance the overall revenue realized from trawling operations. Quality of the shrimps is expected to be better due to minimization of physical pressure caused by accumulation of larger fishes, which is known to take place in conventional codends. The *in situ* sorting effect was very pronounced in the shrimp species and about 97% of the shrimp catch was retained in lower codend. The *in situ* sorting effect and separation of shrimps from finfishes and cephalopods may help to reduce the sorting time and increase useful fishing time of the trawler fishermen and thus enhance the profitability of fishing operations. Increase in towing time can be expected due to slow filling of codend as a result of reduction of non target fishes and juveniles. Fuel saving also can be expected due to drag reduction caused by the escapement of non-target species, from codend.

We have successfully demonstrated installation and operation JFE-SSD to trawler fishermen at fishing villages in Ratnagiri (Maharashtra, India), during 12–14 April 2008, under a collaborative initiative on conservation of trawl caught resources and reduction on the negative impact of trawling on juveniles, of Central Institute of Fisheries Technology (Cochin), College of Fisheries (Ratnagiri) and Cameron International (Mumbai) (Boopendranath et al. 2008). Fishermen did not report any specific difficulties in installation and operation of the device. Enabling policy initiatives and legislation and a rights based regulated access system based on a strong inclusive participatory management seem to be necessary for facilitating large scale adoption of such devices (Boopendranath 2009).

## Conclusions

The JFE-SSD can be easily installed by substituting the conventional codend in a standard shrimp trawl without any alteration in the trawl design. The device reduces bycatch of juveniles of commercially important finfishes, shrimps, crabs and small sized fishes of low commercial value, which will be beneficial for sustainability of resources. The study implies that the device has the potential advantage of retaining larger fishes of higher market value. The *in situ* sorting effect and separation of shrimps from other resources have the potential to reduce the sorting time and increase useful fishing time.

This is the first report of the experimental trials using the newly developed JFE-SSD and the results showed that it has the capacity to pre-sort the catch and favorable bycatch exclusion characteristics which can be beneficially used in the fishery for responsible trawling operations. Further optimization of JFE-SSD, particularly to reduce the finfish catches in lower codend, can be attempted and demonstration of the device in the commercial fishery setting in different fishing areas is required.

## Methods

**Figure 5:**
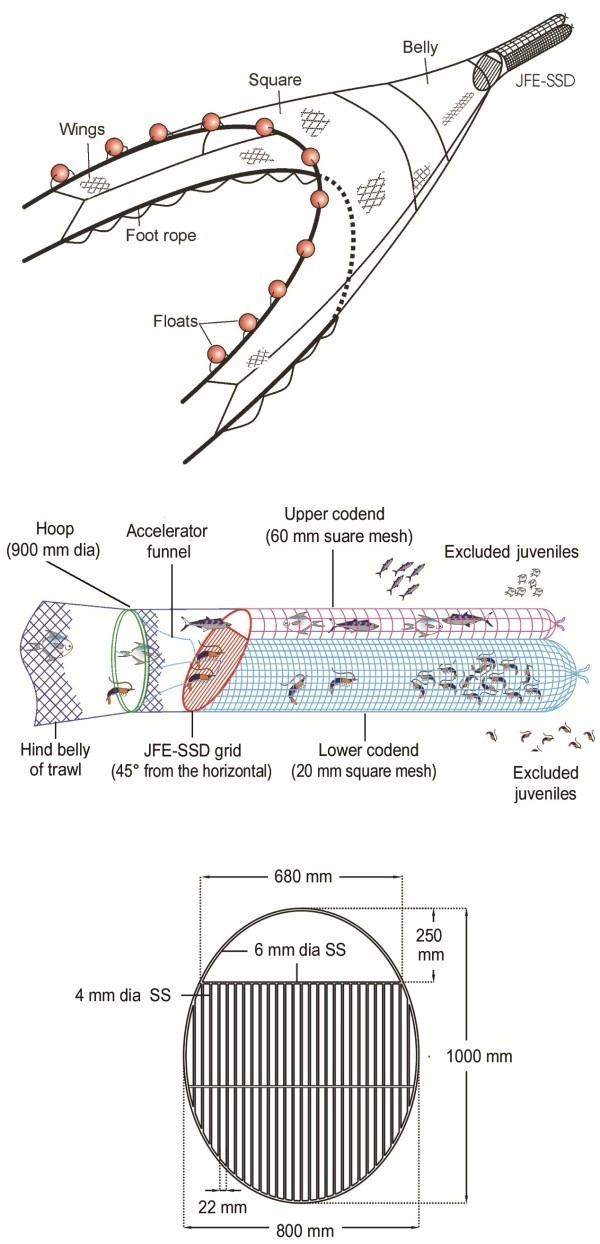
**JFE-SSD: method of installation (top and middle) and design drawing of JFE-SSD grid (bottom).**

Juvenile Fish Excluder cum Shrimp Sorting Device (JFE-SSD) replaces the conventional codend of the shrimp trawl. The device consists of an oval grid made of stainless steel rods having bar spacing of 22 mm kept at 45° angle to the horizontal. The grid is provided with a 250×680 mm top opening which leads to an upper codend with large square meshes (60 mm). A funnel made of netting (20 mm mesh size) guides the catch components towards the lower side of the oval grid kept at 45° angle to the horizontal which separates shrimp from rest of the catch. Shrimps pass through grid bar spacing and are retained in lower codend made up of 20 mm square mesh netting. Juvenile shrimps escape through 20 mm size square meshes of lower codend. The large fishes and cephalopods are deflected upwards to 250×680 mm wide opening provided at the top of the grid and enter into upper codend with large square meshes (60 mm). Juveniles of finfishes and cephalopods and low value small sized finfishes, which have entered upper codend escape through large square meshes (Figure 5).

**Figure 6:**
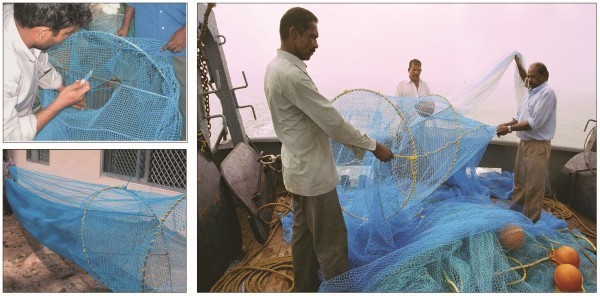
**Views of JFE-SSD under fabrication (upper left), ready for installation (lower left) and its operation (right).**

**Figure 7:**
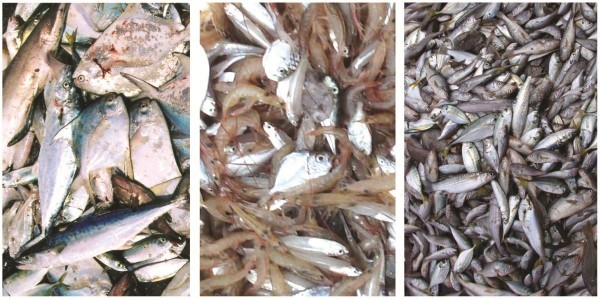
**Views of catch from JFE-SSD operations: upper codend (left), lower codend (middle) and excluded catch (right).**

**Figure 8:**
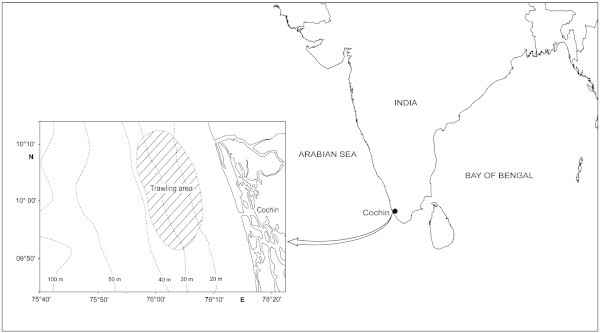
**Fishing area.**

Views of JFE-SSD under fabrication, a finished JFE-SSD ready for installation and its operation are shown in Figure 6 and typical catch from upper and lower codends and excluded catch during JFE-SSD installed operations are given in Figure 7. The experimental field trials using JFE-SSD were conducted off Cochin, south-west coast of India (Figure 8), during November-December 2005 in the traditional shrimp trawling ground off Cochin. The JFE-SSD was fitted to a shrimp trawl of 29.6 m head rope rigged with 87 kg V-form otter boards and operated from Research Vessel *MFB Matsyakumari* (Stern Trawler: 17.5 m L_OA_; 277 hp). Covered codend technique was used for performance evaluation with respect to exclusion from upper codend (Pope et al. 1975; Sparre and Venema 1998). A cover fabricated using 20 mm diamond mesh polyamide netting which was 1.5 times the length and width of upper codend (Stewart and Robertson 1985), was used for retaining the catch excluded from upper codend. No cover was provided in lower codend.

The duration of haul was fixed at 1 hour and after each haul, catches from different codends were sorted and kept separately in trays for length and weight measurements. When shrimp catches were very high, a sub-sample weighing not less than 25% of total weight was used for measurements. The length and weight of each individual was measured to the nearest millimeter and gram respectively. Catches were normalized to CPUE (kg h^-1^) and used for analytical comparisons. Consistency was maintained in deployment and retrieval procedures of the gear during experimental operations to minimize operational errors.

The statistical analysis were carried out using R software (R Development Core Team 2011). One-way ANOVA was used for the comparisons and whenever the data did not follow the assumption of normality, the data was ln + 1 transformed and used for comparisons. The Anderson-Darling procedure was used to compare the length-frequency of species caught in upper and lower codends.

## Abbreviations

JFE-SSD: Juvenile fish excluder cum shrimp sorting device
TL: Total length
CPUE: Catch per unit effort.
